# How informed is consent in vulnerable populations? Experience using a continuous consent process during the MDP301 vaginal microbicide trial in Mwanza, Tanzania

**DOI:** 10.1186/1472-6939-11-10

**Published:** 2010-06-13

**Authors:** Andrew Vallely, Shelley Lees, Charles Shagi, Stella Kasindi, Selephina Soteli, Natujwa Kavit, Lisa Vallely, Sheena McCormack, Robert Pool, Richard J Hayes

**Affiliations:** 1 London School of Hygiene & Tropical Medicine, Keppel Street, London WC1E 7HT, UK; 2 African Medical and Research Foundation, PO Box 1482, Mwanza, Tanzania; 3 National Institute for Medical Research, PO Box 1462, Mwanza, Tanzania; 4 Medical Research Council Clinical Trials Unit, 222 Euston Road, London NW1 2DA, UK; 5 Centre for International Health, University of Barcelona, Spain

## Abstract

**Background:**

HIV prevention trials conducted among disadvantaged vulnerable at-risk populations in developing countries present unique ethical dilemmas. A key concern in bioethics is the validity of informed consent for trial participation obtained from research subjects in such settings. The purpose of this study was to investigate the effectiveness of a continuous informed consent process adopted during the MDP301 phase III vaginal microbicide trial in Mwanza, Tanzania.

**Methods:**

A total of 1146 women at increased risk of HIV acquisition working as alcohol and food vendors or in bars, restaurants, hotels and guesthouses have been recruited into the MDP301 phase III efficacy and safety trial in Mwanza. During preparations for the trial, participatory community research methods were used to develop a locally-appropriate pictorial flipchart in order to convey key messages about the trial to potential participants. Pre-recorded audio tapes were also developed to facilitate understanding and compliance with gel-use instructions. A comprehension checklist is administered by clinical staff to all participants at screening, enrolment, 12, 24, 40 and 50 week follow-up visits during the trial. To investigate women's perceptions and experiences of the trial, including how well participants internalize and retain key messages provided through a continuous informed consent process, a random sub-sample of 102 women were invited to participate in in-depth interviews (IDIs) conducted immediately after their 4, 24 and 52 week follow-up visits.

**Results:**

99 women completed interviews at 4-weeks, 83 at 24-weeks, and 74 at 52 weeks (a total of 256 interviews). In all interviews there was evidence of good comprehension and retention of key trial messages including that the gel is not currently know to be effective against HIV; that this is the key reason for conducting the trial; and that women should stop using gel in the event of pregnancy.

**Conclusions:**

Providing information to trial participants in a focussed, locally-appropriate manner, using methods developed in consultation with the community, and within a continuous informed-consent framework resulted in high levels of comprehension and message retention in this setting. This approach may represent a model for researchers conducting HIV prevention trials among other vulnerable populations in resource-poor settings.

**Trial registration:**

Current Controlled Trials ISRCTN64716212

## Background

HIV prevention trials present a number of unique ethical dilemmas for researchers [1-10]. In many developed and developing country settings, prevention trials are feasible only among vulnerable sub-populations at increased risk of HIV and sexually transmitted infections (STIs) [11,12]. Stigma, income poverty, lack of formal education, illiteracy, and in some at-risk populations, impaired mental state due to alcohol and/or other drug use, mean that obtaining truly informed consent for research participation can be fraught with difficulties in such settings. Vulnerable, desperate subjects are likely to weigh up the potential risks and benefits of trial participation using very different criteria and value judgments compared to less vulnerable subjects [13-17]. Comprehension and retention of key information provided during informed consent procedures, such as overall research objectives or the likelihood and nature of product-related adverse events, may be influenced not only by a subject's educational background or literacy level but also by language barriers and differences in socio-cultural perspectives of health and disease between subjects and the research team [3,14]. Traditional informed consent procedures, based on the use of a written Participant Information Sheet (PIS) and completion of a standardised Informed Consent Form (ICF), are the cornerstone of the International Committee on Harmonization (ICH) Good Clinical Practice (GCP) Guidelines but are now widely recognised to be insufficient alone in obtaining informed consent in such settings [14-16,18]. Innovative approaches to information delivery include the use of an educational video during informed consent for an HIV prevention trial in Port-au-Prince, Haiti, which was associated with high levels of accurate message retention when combined with a face-to-face educational session with a trained study counsellor [18]. Other researchers have affirmed the importance of community engagement in the design of locally-appropriate, culturally resonant informed consent procedures [3,19-21]. The need to consider informed consent as a process (important throughout the period of research participation) rather than a discrete activity (relevant at study entry only) has increasingly been recognised in both developed and developing country settings [14,16,18,22,23].

Mwanza is one of six centres in sub-Saharan Africa participating in the Microbicides Development Programme (MDP), an international partnership for the development of vaginal microbicides for HIV prevention, funded by the UK Department for International Development and Medical Research Council (MRC), and coordinated by the MRC Clinical Trials Unit and Imperial College, London [24]. A feasibility study [25] was carried out among an occupational cohort of women at increased risk of HIV infection and sexually transmitted infections (STIs) in ten administrative wards in Mwanza City, northern Tanzania between July 2002 and March 2005 in preparation for the on-going MDP301 randomized placebo-controlled efficacy and safety trial of the candidate vaginal microbicide PRO2000/5 Gel (Endo Pharmaceuticals, USA), which started in November 2005. Women working in food and recreational facilities, including modern bars, traditional bars (known as *vilabu *or pombe shops in Tanzania), restaurants, hotels, guesthouses, groceries and as informal food vendors (known locally as *mamalishe*), were eligible to participate. Research conducted at a number of sites in Tanzania suggests that some women in this occupational group periodically supplement their income through transactional sex [26,27] and are hence at increased risk of STIs and HIV infection [25,28-31].

In this paper we describe how a participatory, multi-method, continuous informed consent process developed by researchers, study participants and community stakeholders during the preliminary feasibility and pilot phases of the program resulted in high levels of comprehension and message retention in the MDP301 vaginal microbicide trial in Mwanza. This approach may represent a model for researchers conducting HIV prevention trials among other vulnerable populations in resource-poor settings.

## Methods

### Study population

The design of the microbicide trial feasibility study in Mwanza and the baseline socio-demographic, behavioural and biomedical characteristics of study participants have previously been described [25]. In brief, following participatory community mapping to identify eligible food and recreational facilities in ten administrative wards in Mwanza City, a community-based clinic was established in a guesthouse in each ward by October 2002. Study clinics provided free sexual and reproductive health services to participants including voluntary HIV counselling and testing; STI syndromic management; family planning advice and health education. Participants found to be HIV positive were referred to a specialist local public health provider for clinical assessment and care which included antiretroviral provision if appropriate. In addition, an informal referral network of local non-governmental and community-based organizations (NGOs and CBOs) providing care and support for families living with HIV and AIDS was established. Participants with general medical or gynaecological problems were referred to established local care providers for specialist clinical care. A total of 1573 women were enrolled and followed up at three-monthly intervals for up to 24 months. The feasibility study ended in March 2005 with the completion of a small pilot study, conducted among 59 participants, to investigate the acceptability of HPTN035 Placebo Gel in this study population and the feasibility and acceptability of proposed clinical trial procedures [32].

Following the successful completion of the feasibility and pilot studies in Mwanza, the MDP301 efficacy and safety trial commenced among the same study population in November 2005. A total of 1146 HIV sero-negative women were enrolled into the main trial in Mwanza, which completed follow-up in June 2009.

### Community engagement strategies

During the MDP301 feasibility study, a variety of community liaison strategies were developed at the six research centres in Africa. In Mwanza, a participatory approach was adopted and has been described in detail elsewhere [33,34]. Key objectives were to investigate the feasibility of a participatory model of community liaison in this context, facilitated by the use of tools adapted from participatory learning and action techniques (such as listing, scoring, ranking, and diagramming); to establish effective locally-appropriate mechanisms of representation for women participating in the feasibility study; and to build a robust system capable of identifying and responding to key project-related concerns among participants and the broader community. This strategy facilitated open dialogue and two-way communication between researchers and study participants and provided a highly-flexible accessible mechanism through which to disseminate information on research activities at community level. The system was designed around 78 geographical clusters of food and recreational facilities within the ten study wards with representatives at cluster and ward level elected in a process facilitated by the project Community Liaison Officer (CLO, CS). A city-level Community Advisory Committee (CAC) was established with representatives from each ward. Secondary stakeholders representing local public-sector and non-governmental health and social care providers formed a trial Stakeholders' Advisory Group (SAG), which includes two CAC representatives.

The community liaison system was a key integral component of the MDP301 trial in Mwanza and through the network of cluster, ward and city-level representatives provided valuable input to the management of the trial e.g. development and review of locally appropriate standard of care guidelines [35,36].

### Informed consent procedures during the feasibility and pilot studies

**Figure 1:**
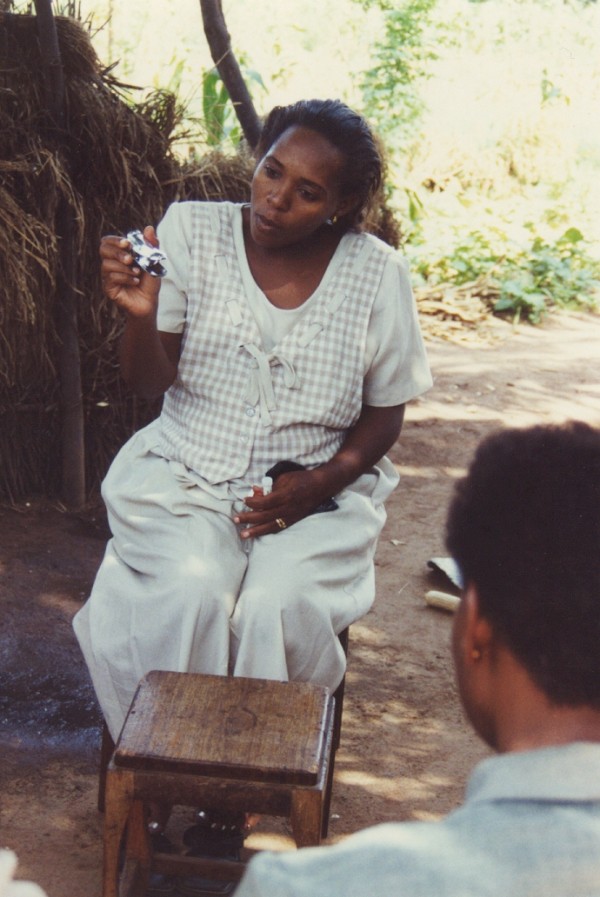
**Fieldworker showing a speculum to women attending a facility-based meeting**.

During the feasibility study in Mwanza, project fieldworkers conducted orientation and mobilisation meetings at facility-level during which women were advised on study objectives, clinical procedures and other study-related issues. Fieldworkers demonstrated the blood collection and clinical examination instruments to be used and passed these around the group so that women could see and handle these items for themselves (Figure 1). Women were encouraged to ask questions about the study and invited to attend an initial clinic visit at which they were advised they would have a more detailed discussion with a member of the clinical team prior to completing formal informed consent procedures (including providing a signature or witnessed thumbprint to confirm their understanding of key study objectives and procedures) and enrolling in the feasibility study. Every participant was given a Participant Information Sheet (PIS) in Swahili and a copy of their completed Informed Consent Form (ICF) to take home.

The relatively high early losses to follow-up observed during the feasibility study[25], coupled with anecdotal feedback from clinic staff and fieldworkers, and information collected from study participants in community participatory workshops, focus group discussions (FGDs) and in-depth interviews (IDIs) [34], suggested that misconceptions and misunderstanding about study objectives were relatively common during the feasibility study and that these may have affected clinic re-attendance.

**Figure 2:**
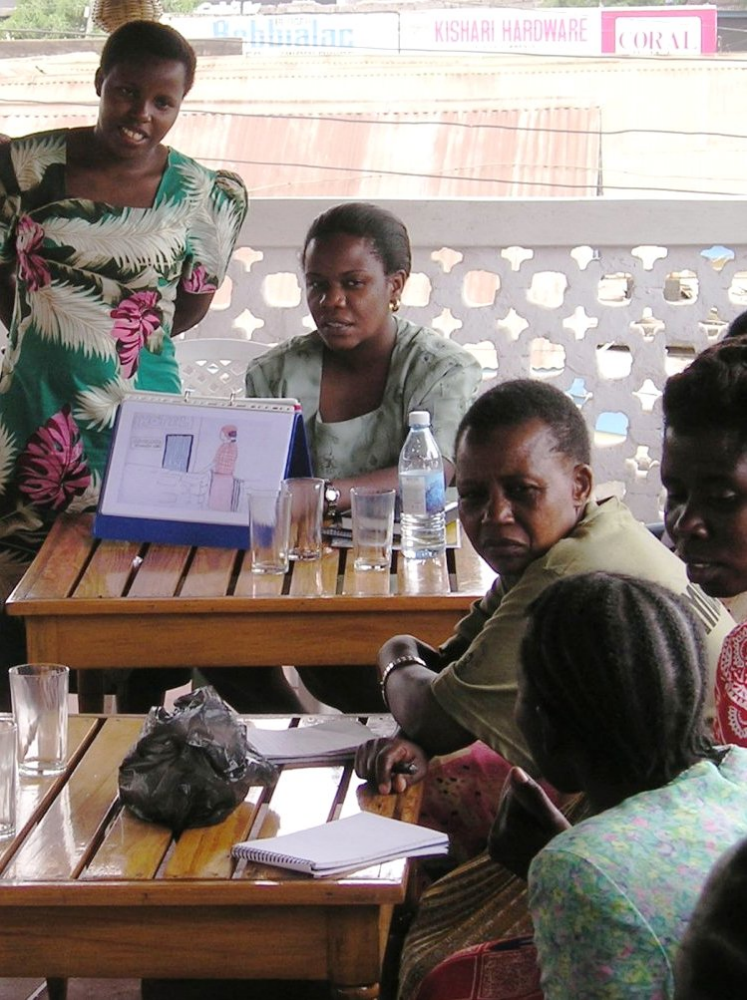
**Reviewing an early version of the pictorial flipchart with CAC members**.

These findings led to a reappraisal of informed consent procedures at the site, culminating in a variety of new approaches being tested in the MDP301 Pilot Study, including a pictorial flipchart developed through consultation with members of the Community Advisory Committee (Figure 2) and subsequently used by project fieldworkers to provide information to potential study participants at facility-based community meetings. Audio tape recordings and pictorial instructions providing information on gel use were also piloted. A site-specific comprehension assessment, which included a qualitative component and a standardised written checklist, was used to evaluate how accurately study participants were able to retain key messages relating to pilot study objectives. IDIs were conducted in order to explore comprehension and message retention in more depth. The high levels of information retention and comprehension observed during the pilot study led to a revised package of informed consent tools and procedures being taken into the main MDP301 phase III clinical trial.

### Informed consent procedures during the MDP301 trial in Mwanza

**Figure 3:**
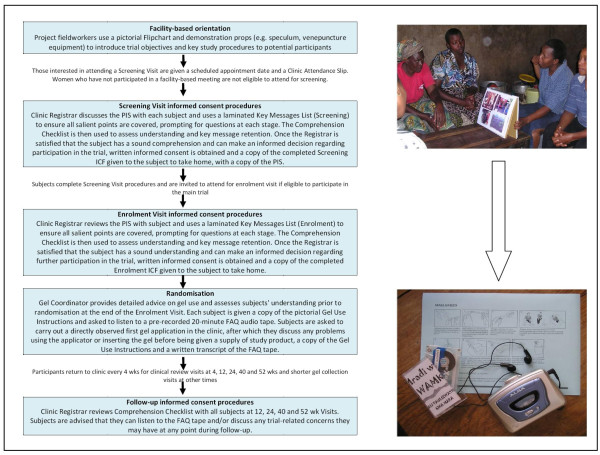
**Continuous informed consent process, MDP301 trial, Mwanza**.

A continuous consent process was developed for the main trial in Mwanza, based on experience gained during the feasibility and pilot study phases of the program, and designed to facilitate accurate and comprehensive message retention from the point of first contact in the community to completion of the final study visit at 52 weeks (Figure 3). Each step in the process required around 30-45 minutes. This approach was facilitated by the sponsors of the MDP301 multicentre phase III trial who provided each trial site with a list of generic key messages to be provided to participants but also strongly encouraged sites to develop locally-appropriate strategies for communicating these messages to participants as part of the informed consent process, in recognition of the diversity of socio-cultural settings within which this research was being undertaken.

**Figure 4:**
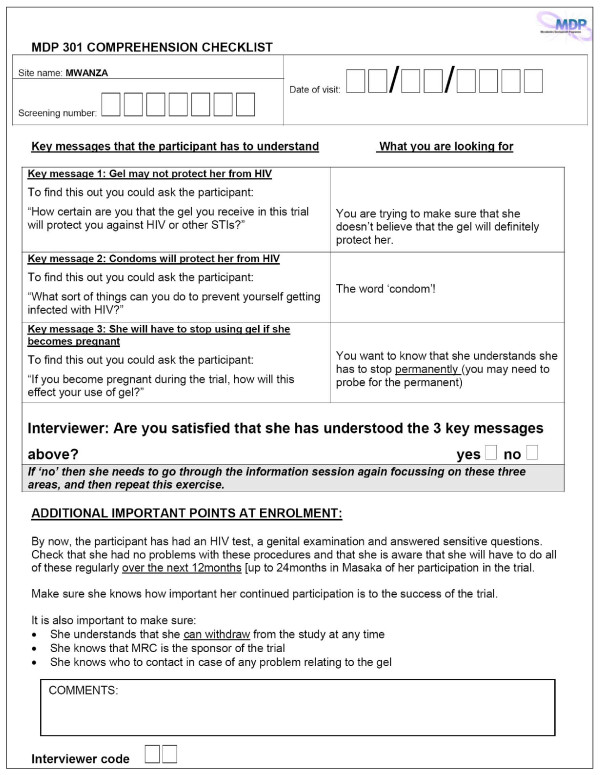
**Comprehension Checklist, MDP301 trial**.

The MDP trial investigators decided to focus on assessing the comprehension of three critical issues: that gel may not protect participants from HIV acquisition; that consistent condom use will prevent HIV infection; and that in the event of pregnancy, gel should be discontinued. An MDP301 trial-specific Comprehension Checklist (Figure 4) was therefore developed and administered by clinical staff to participants at screening, enrolment, 12, 24, 40 and 52 week follow-up visits. Staff provided immediate feedback to participants once the checklist was completed, reviewing any incorrect or unclear responses to each of the three key messages to ensure participants had adequate understanding prior to proceeding with the scheduled visit.

To investigate women's perceptions and experiences of the trial, including how well participants understand and retain key messages provided through a continuous informed consent process, a random sub-sample of 100 women were invited to participate in in-depth interviews (IDIs) conducted immediately after their 4, 24 and 52 week follow-up visits. IDIs evaluated participant understanding and recall as well as assessing comprehension in the context of wider participant perceptions about the trial. This enabled researchers to evaluate message *comprehension *and *retention *but to also investigate message *internalization *in terms of how understanding was incorporated into prevailing beliefs and perceptions of HIV risk, female sexual agency and other issues, such as women's hopes that gel would be effective. IDIs were conducted in Swahili using a semi-structured, standardised interview guide and recorded with the participant's permission. Participants were each asked the same questions relating to the informed consent process at each time point. Transcripts were written in Swahili and translated into English, which were then read and corrected by the original interviewer, in consultation with the Social Science Research Coordinator (SL). Qualitative data were analysed using QSR-Nvivo 2.0 software (QSR International, Australia), using a coding frame developed by the research team, which was supplemented as themes emerged in the course of analysis. IDIs were coded for a variety of key themes in addition to comprehension and informed consent issues, including gel use and acceptability; the acceptability of trial procedures; factors determining condom use; and intravaginal hygiene practices. Data analysis was carried out by the Social Science Research Coordinator (SL) and MDP301 Mwanza Trial Coordinator (AV), who checked their interpretations of the data with the field research team, going back to the Swahili documents for verification where necessary. For each of the three key trial messages in the Comprehension Checklist, participants were categorised into two broad groups based on their responses and comments during in-depth interviews. Simple quantitative univariate analysis was carried out using the Statcalc function of EpiInfo 2000 (CDC, Atlanta, USA).

### Ethical considerations

Ethical clearance for this research was obtained from the National Medical Research Coordinating Committee in Tanzania and the Ethics Committee of the London School of Hygiene and Tropical Medicine in the UK. Pictorial flipcharts, audio tapes, pictorial gel use instructions, comprehension checklists, Participant Information Sheets (PIS) and Informed Consent Forms (ICFs) in English and Swahili were all approved as part of the ethical review process. Written informed consent (signature or witnessed thumbprint) was obtained from all participants at both screening and enrolment visits, in accordance with the International Committee on Harmonization (ICH) Good Clinical Practice (GCP) guidelines. Separate written informed consent for participation in the IDIs was also obtained from each participant.

## Results and Discussion

A total of 256 interviews were carried out, comprising 99 women completing IDIs at 4-weeks, 83 at 24-weeks, and 74 at 52 weeks. The majority of respondents at all time points appeared to have understood and retained key information provided through the informed consent process, including overall trial objectives and messages related to gel, condom use and pregnancy (Table 1). There appeared to be no significant trends in retention or understanding during the period of follow-up.

**Table 1:** Comprehension and retention of key informed consent messages at 4, 24 and 52 weeks follow-up, MDP301 Trial, Mwanza^1^

Message	N (%) of participants with accurate message retention at each visit				Chi square test for linear trend, 4 to 52 weeks (p-value)
		
	Wk 4 (n = 99)	Wk 24 (n = 83)	Wk 52 (n = 74)	All wks combined (N = 256)	
**Key message 1**: Gel may not protect her from HIV	76 (76.8%)	59 (71.1%)	62 (83.8%)	197 (77.0%)	0.90 (p = 0.341)

**Key message 2**: Condoms will protect her from HIV	82 (82.8%)	69 (83.1%)	59 (79.7%)	210 (82.0%)	0.25 (p = 0.619)

**Key message 3**: She will have to stop using gel if she becomes pregnant	92 (92.9%)	78 (94.0%)	69 (93.3%) ^§^	239 (93.3%)	0.19 (p = 0.665)

^1^A single researcher made an assessment of message retention at each time point; it was therefore not possible to calculate a Kappa statistic for concordance

^§ ^There was one missing value at wk 52

### Why is the trial being carried out?

All women interviewed appeared to have a sound basic understanding of why the trial was being conducted, for example:

'They are trying the gel so that they may be sure if it will help to prevent infections of HIV/AIDS'

'I am participating in the study because we want to know if the gel can help us. Not me alone, but that it will benefit all people, even the whole world, the whole of Tanzania'

'They are doing the research because a woman does not have the ability to force a man to use a condom ...this is the reason it has been developed, for a woman to protect herself against HIV'

Complex issues such as the research development process, the concept of a placebo gel and the requirement for randomisation and blinding in the experimental trial design also appear to have been broadly understood by trial participants, despite low levels of literacy and educational attainment among women in this occupational group [25]:

'The gel has gone through research ...three different researches, starting with a research on animals through to a research on human beings. At present it is at the third type of research, they are checking the effectiveness of the gel ...it is being checked to see if it can work in the bodies of people'

*"When we went to the clinic they told us that there were two types of gel, there is a gel with medicine *[jeli yenye dawa] *and a gel without medicine *[jeli bila dawa]*, now for us that use the gel we do not know if it has medicine or not."*

*'The gel is here for the purpose of a research of HIV infection. They test it to see whether it does or does not prevent. It means that if even those who have completed 52 weeks do not know yet whether it has *[effective] *medicine. We too will complete and leave our colleagues participating while it is still under research'*

'I am not sure with gel because we haven't reached the end of the study, we don't know yet if it protects or not'

Many of these issues were explored in depth with community representatives and local stakeholders in Mwanza during participatory community workshops, CAC and SAG meetings during the earlier feasibility and pilot studies [34] in order to appropriately frame these concepts within the consent process (e.g. pictorial representation of randomisation in the site-specific flipchart; appropriate terminology for the placebo gel (*jeli ya placebo; jeli bila dawa *['gel without medicine']) that avoid potentially misleading terms, such as *mafuta *(ointment, oil), which can have overly positive medical overtones). Reinforcing these and other messages from point of first community contact, through screening, enrolment and trial follow-up has been a core principle in Mwanza and may help explain the high levels of trial message comprehension and retention observed among women in this cohort. In addition, the participatory community liaison system (CLS) developed in Mwanza is thought to have facilitated key message reinforcement at facility, cluster and ward level meetings. For example, informal feedback via community representatives and project fieldworkers, supplemented and validated by formal qualitative research methods [34], allowed misconceptions and rumours (*uzushi*) about the trial, its objectives and the risks or benefits of gel use to be captured, and locally-appropriate responses implemented. A number of *uzushi *related to gel were highlighted by community representatives following the start of the MDP301 trial in Mwanza, such as the gel being made from *'white men's sperm' *or that gel *'makes you HIV positive"*. Rumours relating to the purpose of the trial included the belief that the trial was being conducted to allow women to be *"experimented on by white people" *or that *"white people are bringing drugs to kill us"*. Community representatives told us that these *uzushi *were circulating primarily in the broader Mwanza community rather than among women already enrolled in the trial (or those working in food and recreational facilities who would be eligible to enrol), but it is possible that similar views may be held by at least some women in the trial cohort, which is obviously of concern. Data from IDIs conducted during the trial supports the view of community representatives however, and suggests that participants had sufficient knowledge, comprehension and agency to dismiss rumours as untrue or simply irrelevant gossip:

*'Penye wengi hapakosi neno' *[where many gather words never lack]

'As for those rumours, because they can talk and if you don't listen to them, they will talk for two or three days then they will stop.'

Several *uzushi *noted during the feasibility study persisted into the main trial, but appear to be far less widely held in the community and/or been modified to reflect trial-specific procedures. For example, related to the collection of blood specimens, the concern that '*blood might fall into the wrong hands and be sold for witchcraft purposes'*, was prominent among study participants and community members during the feasibility study but have largely disappeared following initiatives undertaken with community representatives and the CAC [34,35]:

'There was a day when I didn't come and a woman started telling them "ee she has gone to give blood, she goes to give blood which is then sold" ...I told her "it is not like they have to take my blood every day I go there, it is every 3 months". After explaining this she asked for forgiveness and now it is finished'

Concerns during the trial have instead re-focussed on the amount of blood collected, as noted by several respondents during IDIs:

'...they are taking too much blood...every time two bottles'

'*...every month you go there you must give blood. Two bottles, in fact such things, according to how I live and my work, you find that there is a problem. You can get sick, and on that day you will have no blood'*

This change of emphasis has occurred within the context of increasing mutual trust and understanding between women enrolled in the trial, potential participants, community members and the research team, established through the community engagement model developed in Mwanza [33,34], but also highlights the limitations of the continuous consent process, since some women remain unclear as to why blood is being collected in the main trial.

### Comprehension and retention of key messages

In the majority of IDIs conducted at all stages during follow-up (Table 1), MDP301 trial participants in Mwanza were able to appropriately recall and articulate key information related to the potential non-protective effect of gel, for example:

*"At present I do not know... until after the results are examined by those experts, they will know and will inform us. But I am hopeful that it *[gel] *can help."*

Among respondents who were asked if they thought gel might protect them against HIV, many respondents appeared to frame their thinking and to have internalised key messages in terms broadly consistent with primary trial outcomes. For example, many women said they were 'unsure' if the gel might be working, reasoning that the trial is still on-going:

*"I can't trust it *[the gel] *so much since it's still on trial"*

"I am not sure about the gel because we haven't reached the end of the study, we don't know yet if it protects or not."

Women also conceptualised the potential benefits of gel use within the context of their individual sexual lives, prevailing socio-cultural contexts and norms. For example, women who engage in commercial and transactional sex in this setting are known to be at increased risk of sexual coercion, gender-based and sexual violence [31]:

"I think the gel can protect against infections, maybe...it can even protect against other infections because you don't get bruises."

*"That lubricant *[gel] *can protect against HIV/AIDS infection because you don't get bruises... they say that this gel doesn't have medicine...but that lubricant can prevent you from getting bruises."*

The effectiveness of consistent condom use for HIV prevention and the need to continue using condoms even in the context of participation in a vaginal microbicide trial appeared to be broadly understood by the majority (>90%, Table 1) of participants:

*"I am not sure *[gel works] *because it's on trial...that's why I use condoms."*

*'I think it *[condoms] *protects but again I am not sure because most of the people are using them but AIDS still continues'*

These findings need to be considered in the light of data obtained during the feasibility and pilot studies in Mwanza [31] and from similar at-risk cohorts in Tanzania [26,28,37,38], which suggest that due to disparate prevailing gender power relations and limited female sexual agency in the context of transactional and commercial sex, high levels of comprehension may translate only partially into sexual behaviours that mitigate risk in this context, as reflected in the relatively low use of condoms during the feasibility study and phase III trial [25]. In addition, several women highlighted locally-prevalent rumours that condoms might be ineffective due to being impregnated with viruses or that they contain holes having been pierced, particularly those available in local stores and other outlets as opposed to those provided by the project:

*'It also protects because the majorities are using condoms although they say that they *[the condoms] *are being pierced. Now how is it pierced if you take it from the shop without holes? They say ooh, others are afraid, I have already checked a condom to see if it has the viruses, if you take it and dry it in the sun you will see the viruses. Now where do those viruses come from while he has taken it from the packet and the packet was intact? But someone will tell you - just try it and see, you buy it from the shop and before you use it, just tear the paper, pull out the condom and put it in clean water or put on a metal sheet, you will see the viruses'*

'There are other condoms that have holes'

In over 90% of IDIs conducted at all stages during follow-up trial participants understood that they should stop using gel if they suspected they might be pregnant.

### How effective was the continuous consent process in Mwanza?

A participatory, multi-method, continuous informed consent process achieved high levels of comprehension and message retention among MDP301 trial participants in Mwanza. This approach was based on experience gained during earlier feasibility and pilot studies and in particular, was informed through a dynamic engagement with community representatives and stakeholders. Our strategy shares a number of common elements with approaches adopted among other vulnerable study populations, namely: a commitment to a continuous consent process comprising the delivery of key information using a variety of different media, at different time points, in a variety of community and clinic-based settings, delivered by staff trained in consent procedures from a variety of different professional and disciplinary backgrounds [18,23]. For example, in the HVTN903 HIV clinical trial in Haiti [18], a short (8-minute) educational video was shown to groups of 5-10 volunteers by a social worker, who then introduced the trial consent form, discussed key elements of the trial and responded to questions. The following day, volunteers met a counsellor for a 30-minute face-to-face discussion about the trial, after which a psychologist conducted further discussion, completed a comprehension form and provided immediate feedback to subjects. Finally, formal written consent was obtained in a subsequent meeting with a trial investigator. In contrast to the Mwanza model, further assessment and reinforcement of key messages during trial follow-up were not part of the approach used in Haiti, nor were participatory community liaison techniques employed to supplement and inform the trial consent process.

These findings are encouraging but several important caveats are worth noting. First, it remains unclear to what extent informed choice to participate in the MDP301 trial in Mwanza was based on a sound understanding of the relative benefits and risks of trial participation or other factors such as altruism, trust, individual priorities (e.g. access to free, high-quality, readily accessible community-based sexual and reproductive health care) or a combination of these potential determinants. As Molyneux et al have pointed out [14], key to this discourse is the conceptual framework within which a decision to participate is framed. For example, are terms used by the research team to describe 'research', 'trial', 'experiment' or 'investigation' (*utafiti, jaribio, uvumbuzi *and *uchunguzi *respectively in Swahili) understood by women within the research conceptual framework used by the research team, or are all such terms considered by the majority of participants within an individual therapeutic conceptual framework and thereby to be synonymous with 'health project' (*mradi wa afya*)? That is when we as researchers talk of trial investigations and procedures during the informed consent process, do volunteers conceptualise these as being individually tailored activities, designed to identity and address their own specific and individual health needs? Given that health service delivery has always been an integral component of the research program in Mwanza [25] and highly regarded by participants, community members and local stakeholders alike [34,35], it is likely that many trial participants may have struggled with these competing constructs as they proceeded through the continuous consent process. Second, the trust and partnership working practices established between researchers and women in the Mwanza occupational cohort through the CLS may actually have *exacerbated *the complexities of informed decision making in this context by increasing the risk of what has become known as 'therapeutic misconception' (i.e. the belief that every aspect of the research has been designed to directly benefit the individual) [14,39,40]. This then pervades an individual participant's ability to truly understand and internalise important trial concepts, particularly the differences between placebo and active gel, and the potential risks of trial participation. Third, a focus on a small number of key themes or messages and in reinforcing these at regular intervals during an HIV prevention trial could potentiate the risk that participants learn appropriate responses by rote rather than facilitate a deeper understanding of trial objectives through questioning and open reasoning during clinic visits or community-based contact with research staff i.e. messages were not appropriately *internalised*. A combination of message reinforcement and open-ended discussion were used throughout the continuous consent process in Mwanza to minimise this risk. Qualitative data from the 256 IDIs completed suggest, as summarised above, that MDP301 trial participants in Mwanza were able to gain a fairly broad understanding of the research process and in particular, were able to conceptualise messages related to trial participation within prevailing local socio-cultural frameworks. A more detailed discussion of these issues and related issues such as women's motivations for trial participation are beyond the scope of this paper but will be explored in depth in a subsequent publication (*S Lees, in draft*).

## Conclusions

A participatory, multi-method, continuous informed consent process developed as part of the MDP301 trial in Mwanza resulted in high levels of comprehension and message retention among MDP301 trial participants in Mwanza. This approach may represent a model for researchers conducting HIV prevention trials among other vulnerable at-risk populations in resource-poor settings.

## Competing interests

The authors declare that they have no competing interests.

## Authors' contributions

All authors contributed to the development and revision of draft manuscripts, read and approved the final version of the manuscript. **AV**: designed and coordinated participatory research, community liaison activities and continuous consent approach in Mwanza (with SL, CS); coordinated and managed fieldwork, including establishing clinic sites and field data collection; coordinated and participated in data collection, analysis and interpretation; conducted a literature review, drafted the manuscript and incorporated revisions into the final version for publication. **SL**: designed and coordinated participatory research, community liaison activities and continuous consent approach in Mwanza (with AV, CS); designed and coordinated qualitative fieldwork (with RP); participated in data collection, analysis and interpretation; assisted in drafting and revising the manuscript. **CS**: designed and coordinated participatory research, community liaison activities and continuous consent approach in Mwanza (with AV, SL); participated in data collection and interpretation; and assisted in drafting and revising the manuscript. **SK**: participated in design and coordination of community liaison activities and continuous consent process in Mwanza; data collection and interpretation; assisted in drafting and revising the manuscript. **SS**: participated in design and coordination of community liaison activities and continuous consent process in Mwanza; data collection and interpretation; assisted in drafting and revising the manuscript. **NK**: participated in design and coordination of community liaison activities and continuous consent process in Mwanza; data collection and interpretation; assisted in drafting and revising the manuscript. **LV**: participated in design and development of continuous consent process in Mwanza; led design and development of pictorial informed consent flipchart; assisted in drafting and revising the manuscript. **SMc**: contributed to design and development of continuous consent process in Mwanza and other MDP sites; participated in data analysis and interpretation; assisted in drafting and revising the manuscript. **RP**: contributed to design and development of continuous consent process in Mwanza and other MDP sites; participated in data analysis and interpretation; assisted in drafting and revising the manuscript. **RH**: contributed to design and development of continuous consent process in Mwanza and other MDP sites; participated in data analysis and interpretation; assisted in drafting and revising the manuscript.

## Pre-publication history

The pre-publication history for this paper can be accessed here:

http://www.biomedcentral.com/1472-6939/11/10/prepub
